# New combination approaches to combat methicillin-resistant *Staphylococcus aureus* (MRSA)

**DOI:** 10.1038/s41598-021-82550-4

**Published:** 2021-02-19

**Authors:** Mohamed H. Sharaf, Gamal M. El-Sherbiny, Saad A. Moghannem, Mohamed Abdelmonem, Islam A. Elsehemy, Ahmed M. Metwaly, Mohamed H. Kalaba

**Affiliations:** 1 Botany and Microbiology Department, Faculty of Science, Al-Azhar University, Cairo, 11884 Egypt; 2 Clinical Laboratory, Stanford Healthcare, San Francisco, USA; 3 Chemistry of Natural and Microbial Products, National Research Centre, Giza, Egypt; 4 Pharmacognosy and Medicinal Plants Department, Faculty of Pharmacy, Al-Azhar University, Cairo, Egypt

**Keywords:** Microbiology, Health care

## Abstract

The herbal products proved to be more promising antimicrobials even though their antimicrobial activity is milder than commercially available antibiotics. Moreover, herbal drugs may act synergistically with antibiotics to kill microbes. In this study, we aimed to enhance the activity of penicillin against MRSA through combination with the active saponin fraction isolated from the *Zygophyllum album* plant. Three different types of metabolites (saponins, sterols, and phenolics) have been extracted from *Zygophyllum album* with ethanol and purified using different chromatographic techniques. The antibacterial activity of crude extract and the separated metabolites were checked against MRSA isolates, Saponin fraction (ZA-S) was only the active one followed by the crude extract. Therefore, the compounds in this fraction were identified using ultra-high-performance liquid chromatography connected to quadrupole time-of-flight mass spectrometry (UHPLC/QTOF-MS) operated in positive and negative ionization modes. UHPLC/QTOF-MS revealed the presence of major six ursane-type tritepenoidal saponins (Quinovic acid, Quinovic acid 3β-O-β-D-quinovopyranoside, Zygophylloside C, Zygophylloside G, Zygophylloside K and Ursolic acid), in addition to Oleanolic acid. Interaction studies between saponin fraction and penicillin against MRSA were performed through the checkerboard method and time-kill assay. According to checkerboard results, only three combinations showed a fractional inhibitory concentration index less than 0.5 at concentrations of (62.5 + 312.5, 62.5 + 156.25, and 62.5 + 78.125 of penicillin and ZA-S, respectively). Time kill assay results showed that the highest reduction in log10 colony-forming unit (CFU)/ml of initial inoculum of MRSA after 24 h occurred by 3.7 at concentrations of 62.5 + 312.5 (µg/µg)/ml of penicillin and ZA-S, respectively. Thus, the combination between saponin fraction of *Zygophyllum album* and penicillin with these concentrations could be a potential agent against MRSA that can serve as possible model for new antibacterial drug.

## Introduction

Drug resistance negatively impacts the health of millions in both developed and developing nations and has a massive financial impact on society^1^. In the current antibiotic crisis, bacterial pathogens are increasingly resistant to all available antibiotic drugs, often through different mechanisms, and to multiple antibiotics in the same organism^2^. Methicillin-resistant *Staphylococcus aureus* (MRSA) constitutes the major threats among antibiotic-resistant agents that cause deaths in the U.S with a health care cost of $3–4 billion annually^3^. Therefore, the infection control and prevention of MRSA remain an important goal for researchers^4^. The antimicrobial products originated from plants presented a promising solution although their antimicrobial action is minor than classic antibiotics. So, herbal compounds alternatively can be used in combination with antibiotics to enhance the activity against bacterial infections^5^. It is necessary to develop new antimicrobial drugs which are effective against MDR microorganisms through the combination of various active agents. This direction of research succeeded to develop therapies which are effective against cancer, HIV, *Mycobacterium tuberculosis* and complicated cases of malaria^6^ There are many positive effects from drug combinations including the treatment of mixed infections, a disease caused by a specific pathogenic organism, limiting the development of resistance and preventing long-term antibiotic use^7^. Here, we have selected the *Zygophyllum album* plant during the screening course for antimicrobials. The plant *Zygophyllum album* is one species of *Zygophyllaceae* that includes about 27 genera and 285 species spreading mostly in the tropical and subtropical areas^8^. *Zygophyllum album* is a widely spread halophyte in Egypt in different areas including North Sinai, Mediterranean Coast and anticlines districts^9, 10^. Halophytes showed a great ability to survive in toxics and high salinity. This interesting ability to resist such biotic stress makes the halophytes a potential source for antimicrobial compounds. Several kinds of secondary metabolites have been isolated from the *Zygophyllum album* including saponins phenolics and steroids^11–14^. Although some reports have been made on *Zygophyllum album*, most of them have not studied the effect of its metabolites on MRSA either individually or in combination with antibiotics. For this reason, this study aims to evaluate the antibacterial activity of these plant metabolites on different MRSA isolates in addition to the effect of interaction between an active fraction of plant metabolite and penicillin as one of the alternative solutions to combat MRSA.

## Materials and methods

### Identification and antibiotic profiling of clinical isolates

Four clinical isolates coded as M-1, M-2, M-3 and M-4 were kindly obtained from the microbiology department at the National Cancer Institute (NCI), Giza, Egypt. These isolates were identified using an automated VITEK2 system^15^. Then, the antibiotic profile of these isolates as well as *Staphylococcus aureus* ATCC 25923 was evaluated using the disc diffusion method on Mueller–Hinton Agar (MHA). MHA was inoculated with each bacterium using sterile cotton swabs. Antibiotic discs representing different classes of antibiotics (Antibiotics panel were cefuroxime 30 μg/ml, metronidazole 5 μg/ml, neomycin 30 μg/ml, tetracycline 30 μg/ml, cefoxitin 30 μg/ml, nalidixic acid 30 μg/ml, clindamycin 2 μg/ml, trimethoprim/sulfamethoxazole 25 μg/ml, ciprofloxacin 5 μg/ml, amoxicillin/clavulanic acid 20/10 μg/ml, gentamycin 10 μg/ml, chloramphenicol 30 μg/ml, bacitracin 10 μg/ml, erythromycin 15 μg/ml, rifampicin 30 μg/ml, kanamycin 30 μg/ml, amikacin 30 μg/ml, rifampin 5 μg/ml and penicillin 10U) were gently loaded on the prepared plates using sterile forceps. The prepared plates were incubated in the refrigerator for 2 h, then incubated at 35 °C for 18 h. The diameter of the inhibition zone was measured in millimeters (mm), compared with the standard zone diameter given in the protocol chart. It can be determined whether the bacterial isolate is resistant, intermediate or susceptible to the tested antibiotics^16^.

### Collection of plant materials and preparation of ethanolic extract

**Figure 1:**
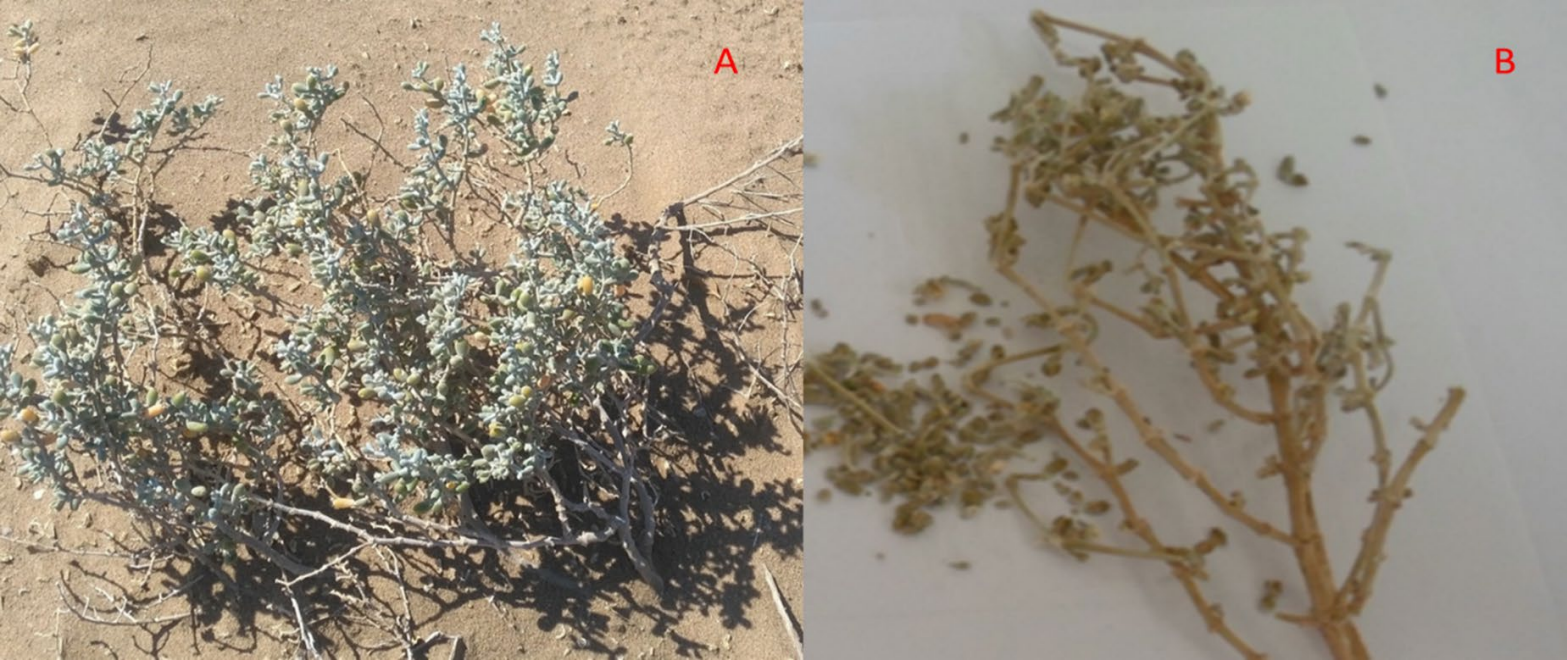
*Zygophyllum album* plant (**A**) and dried aerial part (**B**).

Plant materials Fig. 1 were collected from Bahariya Oasis, Egypt [N 28.22 49.7 E 28.59 10.6] in March 2018. The sampling was done by a randomized collection in an area of about 200 m^2^. Botanical identification of this species was carried out by Dr. El-Baraa Mohammed El-Saied “assistant professor of Plant Ecology, Botany and Microbiology Department, Faculty of Science, Al-Azhar University”.

Ethanolic extract was prepared as follows: 100 g of air-dried aerial part powder of plant was soaked in 1000 ml ethanol (Sigma-Aldrich, St. Louis, MO, USA) in 2500 ml screw-capped bottle, then incubated at room temperature for 48 h on an orbital shaker at 120 rpm (NEW Brunswick scientific Edison, N.J, USA]). The crude extract was obtained by centrifugation at 5000 rpm (SIGMA 2K15) for 10 min and ethanol residue was removed using a rotary evaporator (Heidolph VV2001) to obtain crude extract^17^.

### Separation of different types of plant metabolites

Each group of the major reported secondary metabolites has been isolated and purified using different chromatographic techniques to get three different fractions (saponins, phenolic and steroidal).

### Saponin fraction (ZA-S) isolation

The plant extract was suspended in water, then defatted using ethyl acetate. The defatted extract was shaken with n-butanol saturated with water. The n -butanol aliquots were combined and concentrated under reduced pressure at 60 °C. Crude saponin fraction was precipitated using acetone. The precipitated fraction was further purified using gel filtration chromatography with Sephadex LH-20 column to get pure saponin fraction^18, 19^.

### Phenolic fraction (ZA-P) isolation

The plant extract was suspended in water, then defatted using n-hexane. Then, 25 ml of 1 N HCl were added to the defatted aqueous fraction. The mixture was soaked and maintained at 50 °C for 30 min, then at room temperature for 2 h. The extract was filtered, then neutralized. 50 ml of ether were added to the neutralized filtrate. The ether fraction was separated and allowed to evaporate. The concentrated fraction was purified for the last time using gel filtration chromatography with a Sephadex LH-20 column to extract pure phenolic fraction^20, 21^.

### Steroidal fraction (ZA-St) isolation

The plant extract was mixed with toluene for 12 h on a water bath to remove oils and fats. The residue was hydrolyzed with 2 N HCl (W/V) for 4 h at low temperature (50–60 °C). The mixture was filtered, neutralized with sodium bicarbonate and washed with distilled water until it was neutral (pH 7). The residue was shaken with CHCl_3_ against water. The CHCl_3_ was combined and dried under reduced pressure at 40 °C. The dried CHCl_3_ fraction was saponified according to the prescribed technique^22^. Finally, the un-saponified fraction was purified using a C-18 SPE cartridge to yield pure steroidal fraction^23, 24^.

### Antibacterial assay

Antibacterial activity of the crude extract, saponin, phenolic, and steroidal fractions was tested against the bacterial isolates as well as *Staphylococcus aureus* ATCC 25923 (standard strain)^25^. Briefly, Muller Hinton Agar plates were inoculated with 100 μl of bacterial suspension (10^6^ CFU/ml). Dried paper discs (8 mm) were loaded with 50 µl of both crude extracts, saponin, phenolic, and steroidal fractions at a concentration of (10 mg of each one/ml of DMSO). The loaded paper discs were plated on the surface of the inoculated agar plate. cefoxitin paper disc 30 μg/ml was used as a control antibiotic on the same plates. The loaded plates were incubated at 35 °C for 18 h and the inhibition zone diameter was measured in millimeters (mm). The experiment was performed in three replicates.

### Chemical analysis of main saponins fraction in Zygophyllum album using UHPLC/QTOF-MS

#### Chemicals

LC–MS grade acetonitrile and gradient solvents including isopropanol, methanol, dichloromethane and ethyl acetate were provided by Thermo-Fisher (Thermo Fisher Scientific, USA). Formic acid 98%, ammonium hydroxide, ammonium format and ammonium acetate were purchased from Sigma-Aldrich (Sigma-Aldrich Co. Louis St., MO, USA).

#### Sample preparation

The dried saponin fraction (50 mg) was reconstituted in 1000 µl reconstitution solvent (water:methanol:acetonitrile 2:1:1). The sample was vortexed for 2 min followed by ultra-sonication at 30 kHz for 10 min. Twenty microliters of the stock (50 mg /1000 µl) sample were diluted again with 1000 µl reconstitution solvent and centrifuged at 10,000 rpm for 5 min. The supernatants were transferred into analysis vials. The injected volume was 10 µl with a final concentration of 1 µg/µl. Blank and quality control (QC) samples also underwent LC–MS/MS analysis for quality assurance of the experiment. The sample was injected in both positive and negative modes.

### Instruments and acquisition method

Separation of small molecules was carried out on an Axion AC system (Kyoto, Japan) connected with an autosampler system, an In-Line filter disks pre-column (0.5 µm × 3.0 mm, Phenomenex, USA) and an Xbridge C18 (3.5 µm, 2.1 × 50 mm) column (Waters Corporation, Milford, MA, USA) maintained at 40 °C and a flow rate of 300 μl/min. The mobile phase consisted of solution (A) 5 mM ammonium format in 1% methanol, adjusted to pH = 3.0 using formic acid and solution (B) acetonitrile 100% for the positive mode. While the negative mode solution (C) consisted of 5 mM ammonium format in 1% methanol, adjusted to pH = 8.0 using ammonium hydroxide. The gradient elution was performed with the following program: 0–20 min, 10% B; 21–25 min, 90% B; 25.01–28 min, 10% B; and then 90% B for equilibration of the column.

Mass spectrometry was performed on a Triple TOFTM 5600 + system equipped with a Duo-Spray TM source operating in the ESI mode (AB SCIEX, Concord, Canada). The sprayer capillary and declustering potential voltages were 4500 and 80 eV in positive mode and − 4500 and − 80 V in negative mode. The source temperature was set at 600 °C, the curtain gas was 25 psi, and gas 1 and gas 2 were 40 psi. The collision energy 35 (positive mode) and − 35 (negative mode) V with CE spreading 20 V and ion tolerance 10 ppm were used. Triple TOF 5600 was operated using an information-dependent acquisition (IDA). Batches for MS and MS/MS data collection were created using Analyst TF 1.7.1. IDA method was used to collect full scan MS and MS/MS information simultaneously. The method consisted of high-resolution survey spectra from 50 to 1100 m/z and the mass spectrometer was operated in a pattern where a 50-ms survey scan was detected. Subsequently, the top intense ions were selected for acquiring MS/MS fragmentation spectra after each scan^26^.

### Data processing

MS-DIAL 3.70 software^27^ was used for non-targeting small molecule comprehensive analysis of the sample. According to the acquisition mode, ReSpect positive (2737 records) or ReSpect negative (1573 records) databases were used as reference databases. The search parameters were set as follows: MS1 and MS2 mass tolerance: 0.01 Da and 0.05 Da for data collection, for peak detection; minimum peak height: 100 amplitude, mass slice width: 0.05 Da, smoothing level: 2 scans, minimum peak width: 6 scans, for identification MS1and MS2 tolerance: 0.2 Da/each, for alignment; retention time tolerance: 0.05 min and MS1 tolerance: 0.25 Da. The MS-DIAL output was used to run again on Peak View 2.2 with the Master View 1.1 package (AB SCIEX) for feature (peaks) confirmation from Total Ion Chromatogram (TIC) based on the following criteria; aligned features should have signaled to noise greater than 5 and intensities of the sample: blank should be greater than 5.

### Determination of minimum inhibitory concentrations (MIC)

The MIC values of penicillin (Benzylpenicillin potassium salt, HiMedia Laboratories Pvt. Ltd. India.) and saponin fraction (ZA-S) were determined using microdilution assay in a 96 well plate. Muller Hinton broth medium was inoculated with a cell suspension of MRSA isolates and *Staphylococcus aureus* ATCC 25923 (10^6^ CFU/ml) and 200 µl of the inoculated medium was distributed in each well. Tested compounds (penicillin and ZA-S) were tested in a twofold serial dilution. Penicillin was tested at concentrations ranged from 1000 to 7.81 µg/ml and ZA-S started with 5000 to 39.06 µg/ml. The experiment was performed according to the criteria of M7-A7^28^. Wells containing negative control (medium + penicillin or ZA-S at the tested concentrations) were performed to determine the differences in optical density. The plates were incubated for 18 h at 37 °C and the absorbance was measured at 630 nm. MIC was defined as the lowest concentration of the penicillin or ZA-S which is able to inhibit the visible growth of bacteria.

### Evaluation of penicillin and ZA-S combinations against MRSA using checkerboard method

The stock solution of ZA-S was freshly prepared in DMSO at a concentration of 100 mg/ml. Also, penicillin stock was prepared at a concentration of 10 mg/ml in distilled water. Checkerboard assay was performed using 96 well microplates containing Mueller–Hinton Broth (Difco) with penicillin concentrations which started from MIC to 1/32 × MIC in the columns and ZA-S concentrations which started from MIC to 1/32 × MIC along the rows. There was a combined interaction between penicillin and ZA-S on the plate in a checkerboard style as shown in Table 1. MRSA cells were inoculated at concentration ~ 10^6^ colony-forming unit (CFU)/ml/well.

**Table 1:** Checkerboard matrix of penicillin and ZA-S.

Penicillin							
ZA-S	1	2	3	4	5	6	7
A	MIC + MIC	1/2 + MIC	1/4 + MIC	1/8 + MIC	1/16 + MIC	1/32 + MIC	0 + MIC
B	MIC + 1/2	1/2 + 1/2	1/4 + 1/2	1/8 + 1/2	1/16 + 1/2	1/32 + 1/2	0 + 1/2
C	MIC + 1/4	1/2 + 1/4	1/4 + 1/4	1/8 + 1/4	1/16 + 1/4	1/32 + 1/4	0 + 1/4
D	MIC + 1/8	1/2 + 1/8	1/4 + 1/8	1/8 + 1/8	1/16 + 1/8	1/32 + 1/8	0 + 1/8
E	MIC + 1/16	1/2 + 1/16	1/4 + 1/16	1/8 + 1/16	1/16 + 1/16	1/32 + 1/16	0 + 1/16
F	MIC + 1/32	1/2 + 1/32	1/4 + 1/32	1/8 + 1/32	1/16 + 1/32	1/32 + 1/32	0 + 1/32
G	MIC + 0	1/2 + 0	1/4 + 0	1/8 + 0	1/16 + 0	1/32 + 0	0 + 0

Checkerboard microdilution method was performed in duplicate and evaluated after 24 h of incubation at 37 °C. Wells with no penicillin-ZA-S combinations (only medium with MRSA) were used as growth control. Wells with medium containing the used combinations only (without MRSA) were used as a negative control^29^. The difference in the growth of MRSA was calculated by measuring the optical density at the start and the end of the experiment (after 24 h) using an ELISA plate reader at 630 nm (Mindray MR-96A, China)^30^. The fractional inhibitory concentration index (FICi) explains the interaction between penicillin and ZA-S and is calculated with the following equation:

$${\text{FICi}}\, = \,{\text{FIC}}_{{{\text{penicillin}}\,{\text{antibiotic}}}} \, + \,{\text{FIC}}_{{{\text{ZA}} - {\text{S}}}}$$

where FIC_penicillin antibiotic_ = MIC of penicillin in combination divided by MIC of penicillin alone and FIC _**ZA-S**_ = MIC of ZA-S in combination divided by MIC of ZA-S alone. The results were interpreted as follows: synergism when FICi ≤ 0.5, partial synergy (addition) = FICi > 0.5–≤ 1.0, indifference = FICi > 1–≤ 2.0 while antagonism is considered when FICi > 2^31^.

### Time kill assay

Time kill assay against MRSA cells was conducted to assess the killing potencies of penicillin and ZA-S combinations that showed synergistic action in checkerboard assay. Flasks containing Mueller Hinton Broth medium with penicillin and ZA-S combinations were inoculated with cells of MRSA, at a density of ~ 10^6^ CFU/ml and incubated in a shaker incubator at 120 rpm and 37 °C. Aliquots of each treatment, as well as a positive control (only medium inoculated with the same cell count of MRSA), were withdrawn at time intervals 0, 6, 12, 18, and 24 hands serially diluted in sterile saline solution. Then, 100 µl of each dilution was inoculated onto nutrient agar plates in triplicate. These plates were incubated at 37 °C for 18 h and CFU/ml was counted^29, 32, 33^. The kill measurement and the rate of bacterial death were determined by plotting the viable colony counts as a log10 (CFU/ml) against the time. The interaction was classified as bacteriostatic or bactericidal. Bacteriostatic action was defined as a decrease of < 3 logs CFU/ml and bactericidal effect was defined as a decrease of ≥ 3 log CFU/ml after 24 h of incubation compared with the size of the initial inoculum^34^.

### Electron microscopy study

To investigate the bacterial cell morphology before and after treatments, scanning and transmission electron microscopy studies were performed at the Regional Center of Mycology and Biotechnology, Al-Azhar University, Cairo, Egypt.

#### For scanning electron microscopy (SEM)

Muller Hinton agar medium was prepared with and without the combination of penicillin and ZA-S. MHA medium which contains penicillin and ZA-S combination was prepared at two concentrations which are 62.5 + 312.5 (µg/µg)/ml respectively (lethal dose) and a half of these concentrations (sublethal dose). The prepared media were inoculated with 100 µl of ~ 10^6^ CFU/ml of MRSA and incubated for 18 h at 37 °C. After incubation, a plug of the culture was taken and prepared for examination using SEM (JEOL-JSM-5500LV)^35^.

#### For transmission electron microscopy (TEM)

MRSA cells (100 µl of ~ 10^6^ CFU/ml) were inoculated in Muller Hinton broth amended with the same concentrations of the selected combinations mentioned above. Both controls (only Muller Hinton broth medium inoculated with MRSA at the same cell count) and the treated cells were incubated at 37 °C on a rotary shaker at 120 rpm for 18 h^36^. After incubation, all control and treated cells were prepared and examined using TEM (JEOL 1010)^37^.

### Cytotoxicity studies of a synergistic combination of penicillin and ZA-S

The African green monkey kidney (VERO), Normal human lung fibroblast (MRC-5) and Normal human melanocytes (HBF4) cell lines were obtained from cell culture bank at the holding company for biological product and vaccine (VACSERA), Agouza, Giza, Egypt. The cells were suspended using trypsin/EDTA solution 0.25% and the suspension was adjusted to 5 × 10^4^ cells, then pipetted into 96 well tissue culture plate and incubated for 24 h at 37 °C with 5% CO_2_^38^. Different concentrations were prepared in a series of double-fold dilutions starting from 500 and 625 µg/ml for penicillin and ZA-S respectively followed by incubation for 72 h at 37 °C and 5% CO_2_. The cytotoxic effect of treatments that exhibit morphological abnormalities was observed using a Carl Zeiss ID03 inverted microscope (GmbH, Germany). The cytotoxicity was measured using a 3-(4,5-dimethylthiazol-2-yl)-2,5-diphenyltetrazolium bromide (MTT) assay^39^. MTT solution (100 µl/well, 0.5 mg/ml) was added and the plate was incubated in dark for 4 h. The formed crystals of formazan were dissolved in DMSO (100 µl/well) and the absorbance was measured at 570 nm. The concentration of combination required for 50% of cell inhibition (IC-50 value) was calculated, then the cytotoxicity was expressed in terms of cell viability percentage^40^.

### Statistical analysis

In a time-kill study, to determine the differences (*p*-value ≤ 5) in the growth of MRSA (control and treated with combinations) during all time intervals, data was analyzed using a two-way model of analysis of variance with repeated measure (ANOVA-RM). One-way analysis of variance (ANOVA) was used to calculate the differences among the used concentrations in cytotoxicity study using Minitab 18 software extended with a statistical package and Microsoft Excel 365.

## Results

### Identification and antibiotic profile of the clinical isolate

The clinical isolates were identified using the vitek2 automated system as *Staphylococcus aureus* with a very good probability of 93% for M-1, M-2, and M-3 isolates and a probability of 95% for M-4 isolate. The antibiotic susceptibility of these isolates was determined using 19 antibiotics representing different classes of antibiotics. Generally, the results indicated the widespread emergence of multidrug resistance among the tested isolates, where M-1, M-2, M-3 and M-4 were resistant to cefuroxime, metronidazole neomycin, cefoxitin, nalidixic acid, kanamycin and penicillin. The antibiotic profile of tested isolates was varied depending on the resistance of these isolates to the used antibiotics; for example, M-1, M-2, M-3, and M-4 were resistant to 14, 13, 12 and 18 antibiotics respectively among the tested 19 antibiotics. *Staphylococcus aureus* ATCC-25923 that used as control strain was appeared as susceptible to the majority of tested antibiotics Table 2.

**Table 2:** Antibiotic profile of bacterial isolates and *S. aureus* ATCC-25923.

Isolates	Antibiotic	Cefuroxime30 μg/ml	Metronidazole 5 μg/ml	Neomycin 30 μg/ml	Tetracycline 30 µg/ml	Cefoxitin 30 µg/ml	Nalidixic acid 30 μg/ml	Clindamycin 2 µg/ml	Trimethoprime/Sulfo- methoxazole 25 µg/ml	Ciprofloxacin 5 µg/ml	Amoxicillin/Clavulanic acid 30 μg/ml	Gentamycin 10 µg/ml	Chloramphenicol 30 µg/ml	Bacitracin 10 µg/ml	Erythromycin 15 µg/ml	Rifampicin 30 µg/ml	Kanamycin 30 μg/ml	Amikacin30 µg/ml	Rifampin 5 μg/ml	Penicillin 10 U
	Abb	CXM	MET	N	TE	FOX	NA	DA	SXT	CIP	AMC	CN	C	B	E	RF	K	AK	RA	P
M-1	I.Z. D	0	0	12	0	0	0	18	0	0	0	0	22	19	0	0	0	15	25	0
	Interpretation	R	R	R	R	R	R	I	R	R	R	R	S	S	R	R	R	I	I	R
M-2	I.Z. D	0	0	0	8	0	0	19	0	0	0	0	24	14	22	23	0	14	23	0
	Interpretation	R	R	R	R	R	R	I	R	R	R	R	S	S	I	I	R	R	I	R
M-3	I.Z. D	0	0	0	28	0	0	0	15	14	25	0	23	13	14	0	0	0	0	0
	Interpretation	R	R	R	S	R	R	R	I	I	S	R	S	I	I	R	R	R	R	R
M-4	I.Z. D	0	0	0	10	0	0	0	0	0	0	18	0	0	0	17	0	13	13	0
	Interpretation	R	R	R	R	R	R	R	R	R	R	S	R	R	R	R	R	R	R	R
*S.aureus* ATCC-25923	I.Z. D	33	0	10	20	25	0	28	32	24	32	21	26	18	27	21	25	22	29	34
	Interpretation	S	R	R	S	S	R	S	S	S	S	S	S	S	S	I	S	S	S	S

Abb., abbreviation; I.Z.D., inhibition zone diameter; Int., interpretation; R, resistant; I, intermediate; S, susceptible.

### Antibacterial activity of *Zygophyllum album* crude extract and separated fractions

**Figure 2:**
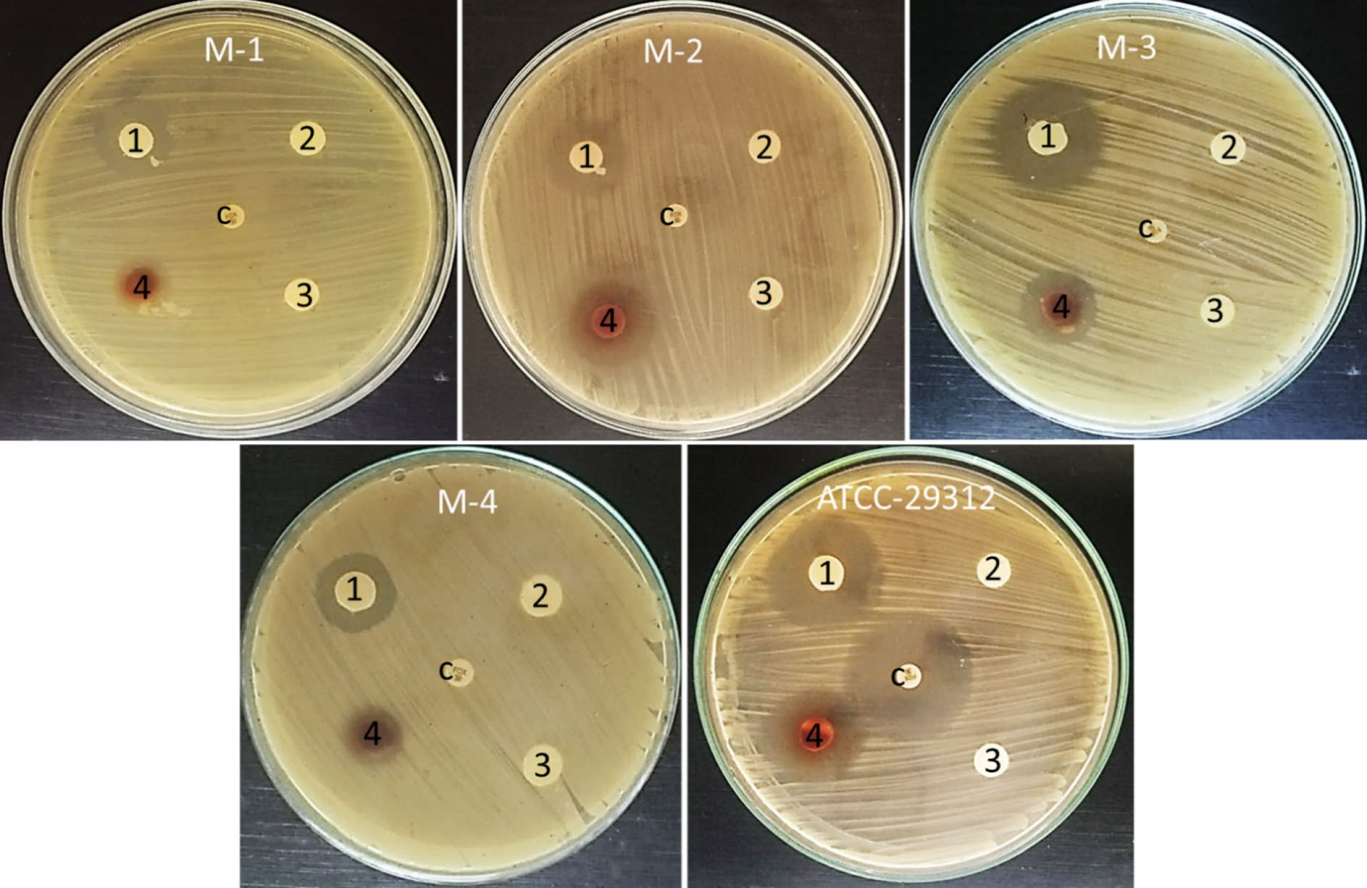
Antibacterial activity of saponin fraction (1), Phenolic fraction (2), Steroidal fraction (3), Crude extract (4), and cefoxitin (C) against MRSA isolates and *Staphylococcus aureus* ATCC 25923.

The separated saponins, phenolic and steroidal fractions as well as crude extract of *Zygophyllum album* were screened for their antibacterial potential against MRSA isolates and *Staphylococcus aureus* ATCC 25923. *Zygophyllum album* crude extract was active against MRSA isolates and *Staphylococcus aureus* ATCC 25923 and showed inhibition zone diameter which ranged from 12 to 22 mm. Both phenolic and steroidal fractions not did not show any effect on all tested bacteria as well as standard strain. On the other side, the saponin fraction was active on MRSA isolates and *Staphylococcus aureus* ATCC 25923 and showed an inhibition zone which started from 16 to 26 mm depending on the tested bacteria. Cefoxitin control antibiotic had no inhibition activity on MRSA isolates, but it showed inhibition zone diameter (29 mm) against *Staphylococcus aureus* ATCC 25923 Table 3 and Fig. 2.

**Table 3:** Antibacterial activity of *Zygophyllum album* crude extract and its fractions.

Isolates	Mean of inhibition zone diameter (mm) ± standard error				
	Saponin fraction	Phenolic fraction	Steroidal fraction	Crude extract	Cefoxitin
M-1	18 ± 1.66	0	0	12 ± 0.66	0
M-2	18 ± 1.15	0	0	14 ± 0.66	0
M-3	26 ± 0.33	0	0	18 ± 0.88	0
M-4	16 ± 0.88	0	0	14 ± 0.57	0
*S. aureus* ATCC-25923	25 ± 0.88	0	0	22 ± 0.66	29 ± 1.45

### Chemical structures of the main identified saponins in *Zygophyllum album* using UHPLC/QTOF-MS

The promising anti-MRSA activity of saponin fraction drove us to identify the chemical composition in this fraction. Consequently, the saponin fraction of *Zygophyllum album* was analyzed by reversed-phase UHPLC/QTOF-MS using a gradient mobile phase in an order of decreasing polarity.

**Figure 3:**
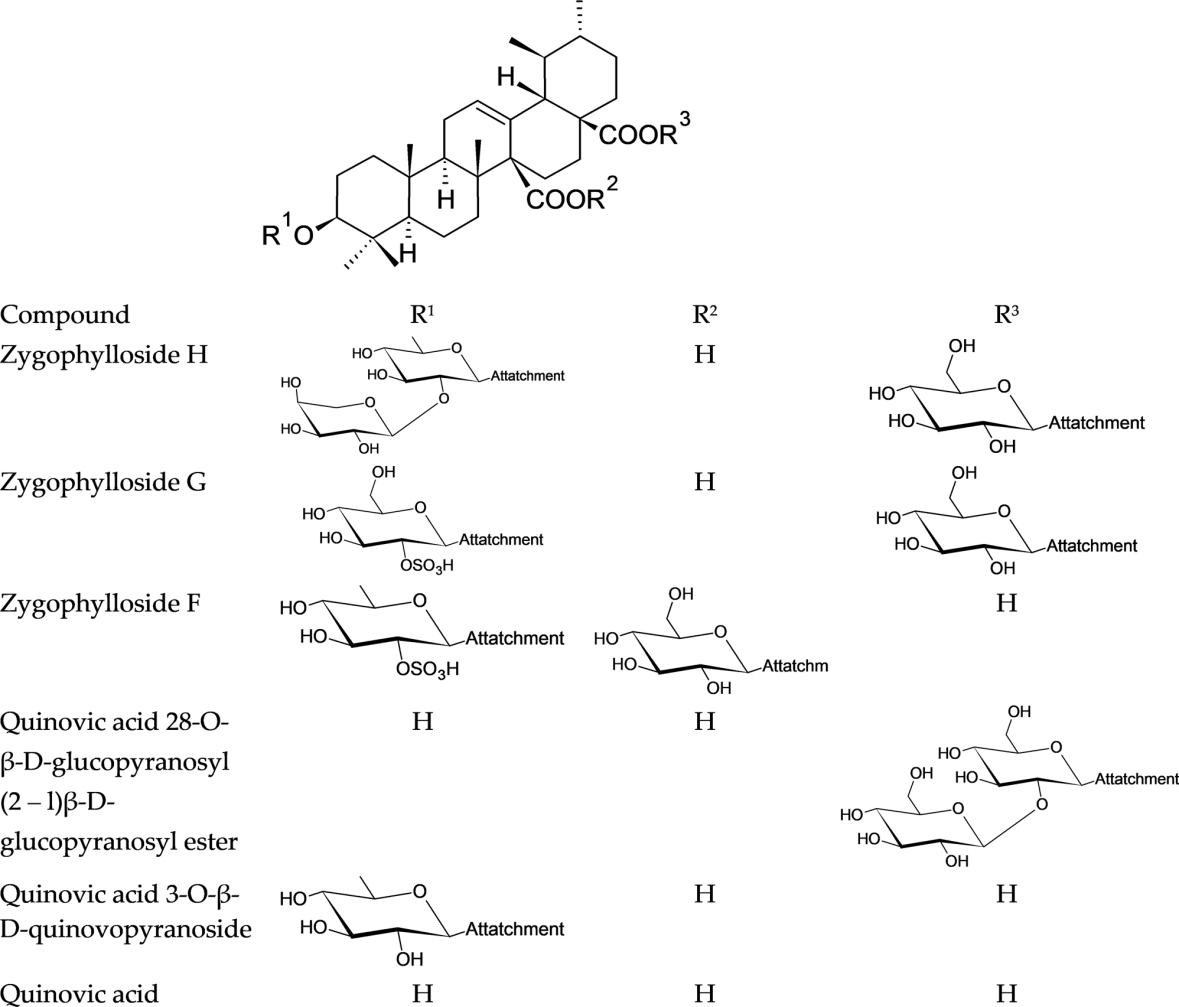
Chemical structures of the main identified saponins in the saponin fraction of *Zygophyllum album*.

UHPLC/QTOF-MS revealed the presence of seven ursane-type tritepenoidal saponins (zygophylloside H, zygophylloside G, zygophylloside F, quinovic acid 28-O-β-D-glucopyranosyl(2–l)β-D-glucopyranosyl ester, Quinovic acid 3-O-β-D-quinovopyranoside, quinovic acid and ursolic acid) as shown in Fig. 3.

### 3-O-[α-L-arabinopyranosyl-(1–2)-β-D-quinovopyranosyl]-quinovicacid-28-O-[β-D-glucopyranosyl]ester (zygophyloside H)

The negative liquid secondary-ion mass spectrum of zygophyloside H showed a quasimolecular ion at m/z 925.4829, [M-H]− proposing C_47_H_74_O_18_ as a molecular formula. This formula was further confirmed by the positive liquid secondary-ion mass spectrum which showed a pseudomolecular ion at m/z 949.4888, [M + Na]+. The identification was consistent with the diagnostic fragment ion at m/z 763.4293, [M-1-162]− corresponding to the elimination of a glucose unit from the deprotonated molecular ion.

### 3-O-[β-D-2-O-sulphonylglucopyranosyl]-quinovic acid-28-O-[β-D-glucopyranosyl] ester (Zygophyloside G)

Zygophyloside G negative liquid secondary-ion mass spectrum showed a quasimolecular ion at m/z 889.3933, [M-H]− proposing C_42_H_66_O_18_S as a molecular formula. This formula was further confirmed by the positive liquid secondary-ion mass spectrum which showed a pseudomolecular ion at m/z 891.4041, [M + H]+ . The chemical structure was authenticated by the diagnostic fragment ions at m/z 727.3401 [M-1–162]− and 683.3498 [M-1–162-44] −showing the sequential loss of a glucose moiety and a glucose moiety plus CO_2_ from the deprotonated molecular ion. The –OSO_3_H group presence was confirmed by the fragment ion at m/z 97.

### 3-O-[β-D-2-O-sulphonylquinovopyranosyl]-quinovic acid- 27-O-[β-D-glucopyranosyl] ester (zygophyloside F)

The molecular formula C_42_H_66_O_17_S was confirmed for zygophyloside F due to the presence of a quasimolecular ion at m/z 873.4034, [M-H]− in the negative liquid secondary-ion mass spectrum.

The chemical structure was consistent with the molecular formula due to the presence of diagnostic fragment ions at m/z 711 [M-1–162]− and 587 [M-1–162-44–80]− indicating the loss of a glucose moiety, then a glucose moiety plus CO_2_ plus SO_3_ from the deprotonated molecular ion.

### Quinovic acid 28-O-β-D-glucopyranosyl (2–l)β-D-glucopyranosyl ester

The positive liquid secondary-ion mass spectrum showed a pseudomolecular ion at m/z 833.4312, [M + Na]+ indicating C_42_H_66_O_15_ as a molecular formula. The proposed structure was consistent with the diagnostic fragment ions at m/z 671.3789, [M + 23–162]+ and 653.3689, [M + 23–162–18]+ corresponding to the elimination of a glucose unit and glucose unit plus water from the pseudo molecular ion.

3-O-[β-D-quinovopyranosyl]-quinovic acid:

Negative liquid secondary-ion mass spectrum showed a quasimolecular ion at m/z 631.3911, [M-H]-proposing C_36_H_56_O_9_ as a molecular formula. This formula was further confirmed by the positive liquid secondary-ion mass spectrum which showed a pseudomolecular ion at m/z 633.4017, [M + H]+. The diagnostic fragment ion at m/z 485.3267, [M-1–146]− suggested the loss of a deoxyhexose unit (quinovose sugar) from the deprotonated molecular ion.

### Quinovic acid and Ursolic acid

The presence of quinovic acid and ursolic acid was confirmed according to their negative and positive liquid secondary-ion mass spectra Table 4.

**Table 4:** Peak annotations of major saponins in *Zygophyllum album* using UPLC- QTOF-MS in negative and positive ionization modes.

Peak	Compound name	Chemical formula	RT (min)	Adduct Ion (− ve mode)	Error (ppm)	Adduct Ion (+ ve mode)	Error (ppm)	Diagnostic fragments (*m/z*)	Ref.
1	Zygophylloside H	C_47_H_74_O_18_	11.3	[M-H]^+^ 925.4829	3.56	[M + Na]^+^ 949.4888	12.11	763.4293, [M-H- gluc.]^−^	^41^
2	Zygophylloside G	C_42_H_66_O_18_S	12.1	[M-H]^−^ 889.3933	4.72	[M + H]^+^ 891.4041	0.78	727.3401 [M-H- gluc.]^−^ 683.3498 [M-H- gluc.-Co_2_]^−^	^41^
3	Zygophylloside F	C_42_H_66_O_17_S	14.3	[M-H]^−^ 873.4034	10.53	–	–	711.3414 [M-H- gluc.]^−^587.3947 [M-H- gluc.-Co_2_-SO_3_]^−^	^42^
4	Quinovic acid 28-O- β-D-glucopyranosyl (2–l)β-Dglucopyranosyl ester	C_42_H_66_O_15_	15.1	–	–	[M + Na]^+^ 833.4312	1.55	671.3789 [M + Na-gluc.]^+^ 653.3689 [M + Na-gluc.-H_2_O]^+^	^13^
5	Quinovic acid 3-O-β-D-quinovopyranoside	C_36_H_56_O_9_	17.4	[M-H]^-^631.3911	10.29	[M + H]^+^ 633.4017	2.36	485.3267	^42^
6	Quinovic acid	C_30_H_46_O_5_	19.8	[M-H]^−^ 485.3301	7.01	[M + Na]^+^ 509.3252	1.96		^12^
7	Ursolic acid	C_30_H_48_O_3_	23.2	[M-H]^−^ 455.3539	3.07	[M + H]^+^ 457.3712	6.77		^43^

### Minimum inhibitory concentrations (MIC) of ZA-S and penicillin

The MIC of both ZA-S and penicillin were determined against MRSA isolates and *Staphylococcus aureus* ATCC 25923 using the microdilution method. The highest MIC value of penicillin (250 μg/ml) was observed with three isolates of MRSA which were M-1, M-2, and M-4, while M-3 was inhibited with MIC 125 μg/ml. MIC values of ZA-S were varied depending on the tested bacteria. The highest MIC was observed with M-1 and M-4 (1250 μg/ml), while the lowest MIC was observed with M-3 (312.5 μg/ml). Penicillin and ZA-S have inhibited the growth of *Staphylococcus aureus* ATCC 25923 at MIC values of 31.25 and 156.25 µg/ml respectively as listed in Table 5.

**Table 5:** Minimum inhibitory concentration (MIC) of ZA-S and penicillin against bacteria.

Isolates	Minimum inhibitory concentration (μg/ml)	
	Penicillin	ZA-S
M-1	250	1250
M-2	250	625
M-3	125	312.5
M-4	250	1250
*S. aureus* ATCC 25923	31.25	156.25

According to the previous data including antibiotic profiling of MRSA isolates, antibacterial activity and MIC determination, M-4 isolate was selected as a model for further studies.

### Synergistic effects of penicillin and ZA-S combination based on FIC index against MRSA M-4

In checkerboard assay, **t**he interactions between penicillin and ZA-S against MRSA M-4 exhibited twenty-two treatments causing inhibition of MRSA M-4. A synergistic effect was considered when penicillin and ZA-S combination showed FICi value ≤ 0.5, this case was observed with only three combinations at different ratios (62.5 + 312.5, 62.5 + 156.25 and 62.5 + 78.125 (µg/µg)/ml of penicillin and ZA-S, respectively). Also, there were eight combinations with FICi ranged from 0.53 to 1 meaning additive effects. On the other hand, eleven combinations showed an indifferent effect where the FICi ranged from 1.03 to 2. The FIC indexes for the tested combinations and their interpretations are presented in Table 6.

**Table 6:** Checkerboard results of penicillin and ZA-S combinations against MRSA M-4.

No	Penicillin/ZA-S ratio	Penicillin/ZA-S ratio (µg/ µg)/ml	Penicillin FIC	ZA-S FIC	FICi	Reaction
1	MIC + MIC	250 + 1250	1	1	2	Indifferent
2	MIC + 1/2	250 + 625	1	0.5	1.5	Indifferent
3	MIC + 1/4	250 + 312.5	1	0.25	1.25	Indifferent
4	MIC + 1/8	250 + 156.25	1	0.125	1.125	Indifferent
5	MIC + 1/16	250 + 78.125	1	0.0625	1.0625	Indifferent
6	MIC + 1/32	250 + 39.06	1	0.03125	1.0312	Indifferent
7	MIC + 0	250 + 0	1	0	1	Additive
8	1/2 + MIC	125 + 1250	0.5	1	1.5	Indifferent
9	1/2 + 1/2	125 + 625	0.5	0.5	1	Additive
10	1/2 + 1/4	125 + 312.5	0.5	0.25	0.75	Additive
11	1/2 + 1/8	125 + 156.25	0.5	0.125	0.625	Additive
12	1/2 + 1/16	125 + 78.125	0.5	0.0625	0.5625	Additive
13	1/2 + 1/32	125 + 39.06	0.5	0.03125	0.5312	Additive
14	1/4 + MIC	62.5 + 1250	0.25	1	1.25	Indifferent
15	1/4 + 1/2	62.5 + 625	0.25	0.5	0.75	Additive
16	1/4 + 1/4	62.5 + 312.5	0.25	0.25	0.5	Synergism
17	1/4 + 1/8	62.5 + 156.25	0.25	0.125	0.375	Synergism
18	1/4 + 1/16	62.5 + 78.125	0.25	0.0625	0.3125	Synergism
19	1/8 + MIC	31.25 + 1250	0.125	1	1.125	Indifferent
20	1/16 + MIC	15.6 + 1250	0.0625	1	1.0625	Indifferent
21	1/32 + MIC	7.8 + 1250	0.03125	1	1.0312	Indifferent
22	0 + MIC	0 + 1250	0	1	1	Additive

### Time kill assay

**Figure 4:**
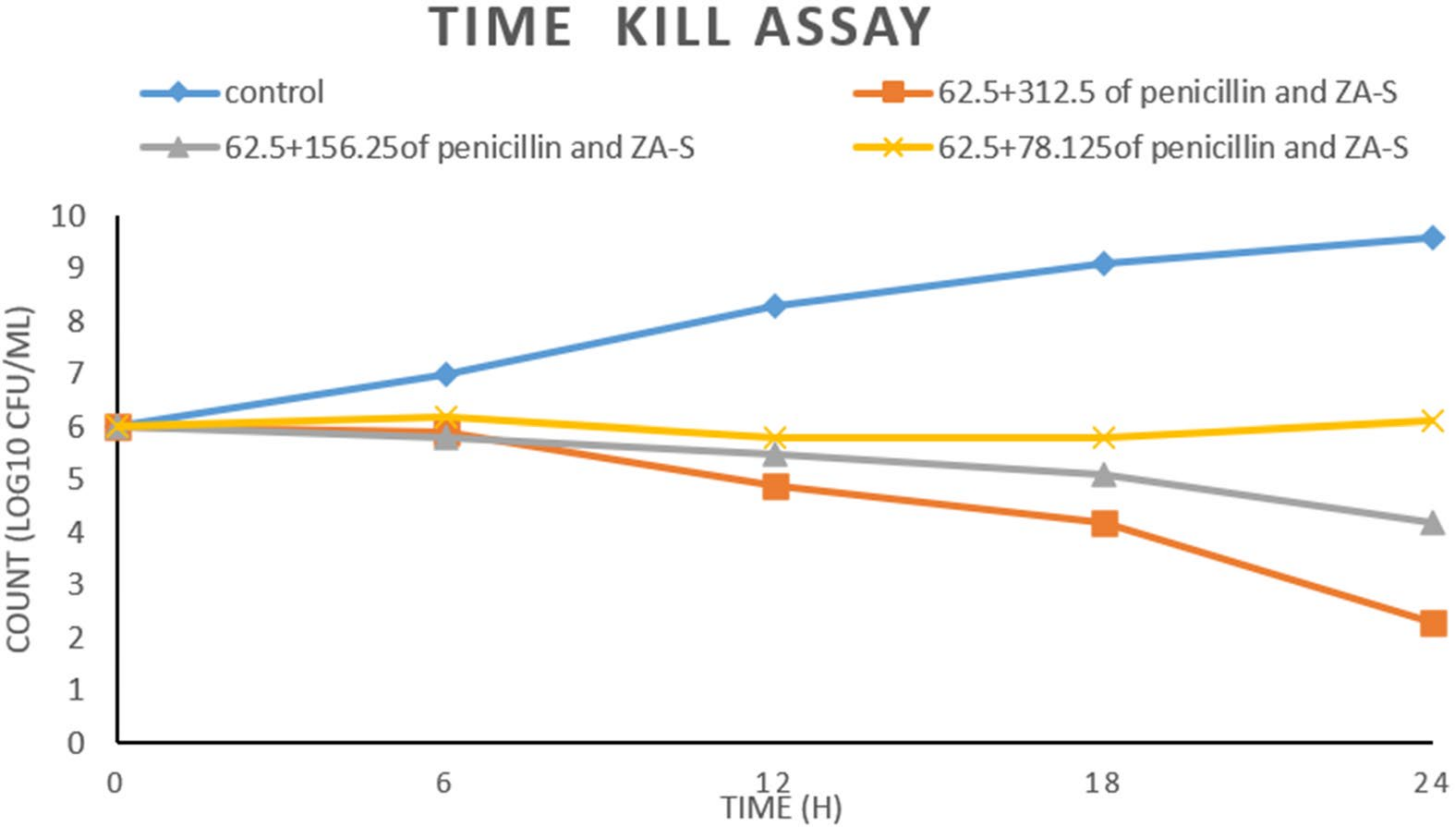
Time kill assay of synergistic combinations of penicillin and ZA-S against MRSA M-4.

The data represented graphically in Fig. 4 refers to the inhibitory effect of different combinations (62.5 + 312.5, 62.5 + 156.25 and 62.5 + 78.125 (µg/µg)/ml of penicillin and ZA-S, respectively) on the growth of MRSA M-4. All treated cultures were affected in a concentration-dependent manner which means that the reduction in CFU count of MRSA M-4 was increased by increasing the concentrations of ZA-S in each combination in comparison with the initial inoculum. Positive control reflects the ideal growth behavior of MRSA M-4 during 24 h of incubation. The combination 62.5 + 312.5 (µg/µg)/ml of penicillin and ZA-S respectively did not allow the CFU count of MRSA M-4 to increase from the onset of the experiment to its end; it significantly reduced the CFU count of initial inoculum during all time intervals of the experiment especially after 24 h of incubation where the reduction of CFU count was (− 3.7). Also, the combination at 62.5 + 156.25 (µg/µg)/ml of penicillin and ZA-S respectively reduced the CFU count after 24 h by (− 1.8). On the other hand, the combination at 62.5 + 78.125 suppressed the growth of MRSA M-4 for 18 h only. After that, it was regrown until reached (6.1) which means an increase by (0.1) compared to the CFU count of the initial inoculum.

### Electron microscopy studies

**Figure 5:**
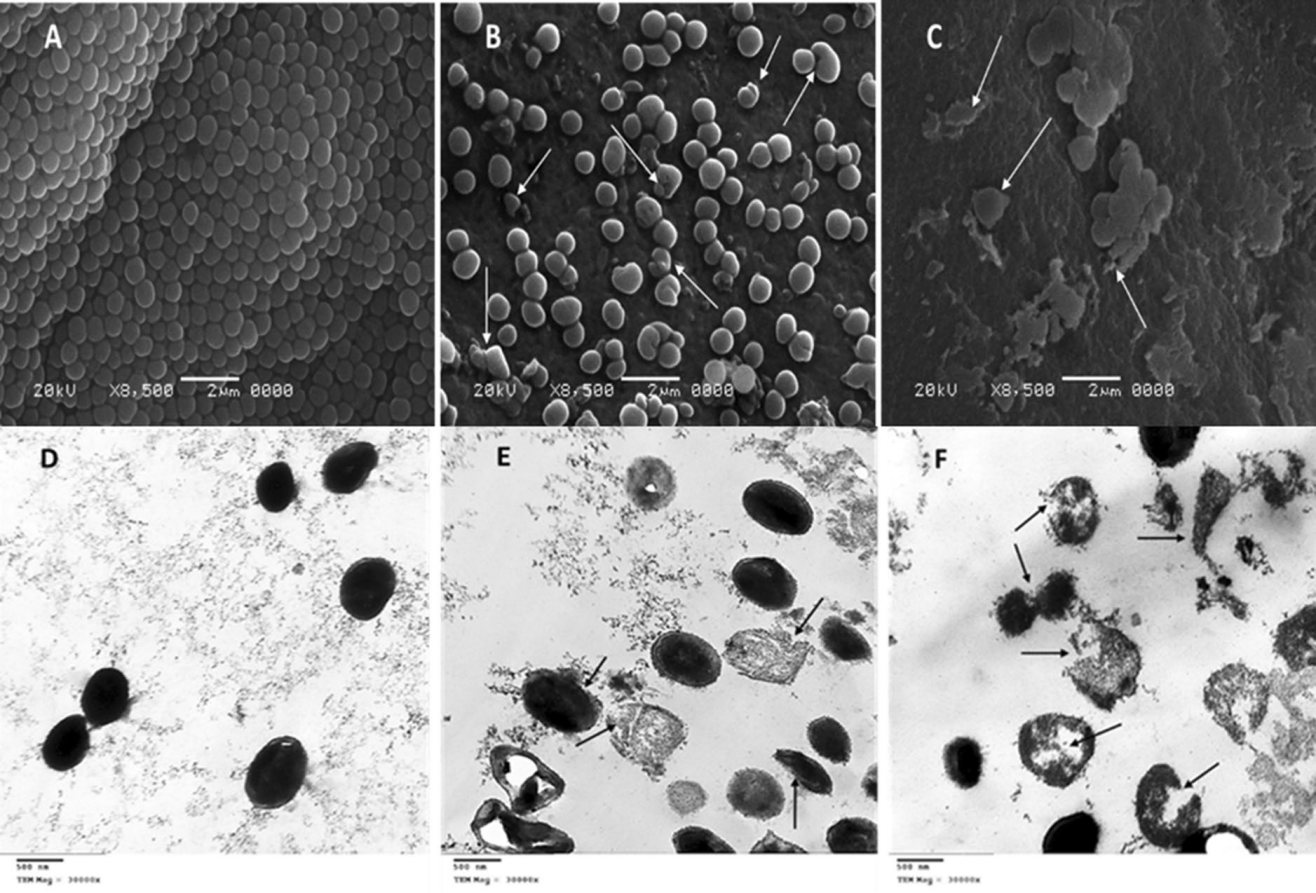
SEM micrographs of MRSA M-4 cells: (**A**) micrograph of untreated cells (**B**) treated cells with sublethal dose (half MIC) of penicillin-ZA-S combination (**C**) treated cells with a lethal dose (MIC) of the penicillin-ZA-S combination. TEM micrographs of MRSA M-4 cells: (**D**) micrograph of untreated cells (**E**) treated cells with half MIC of penicillin-ZA-S combination. (**F**) Treated cells with MIC of the penicillin-ZA-S combination.

The obtained SEM micrographs of untreated MRSA M-4 showed normal morphology, where they were typically round-shaped, with smooth and intact cell surface Fig. 5A. Most treated cells with a combination of penicillin and ZA-S at half MIC concentration (sublethal dose) showed deformed morphology, whereas the cells slightly increased in their size and showed irregular shape, and other cells had a wrinkled surface with some appendages like buds Fig. 5B. The cells treated with MIC concentration (lethal dose) displayed several apparent, distinguished signs of cell damage, including missing the walls or breaking them which led to distorted shape. The cell membrane was progressively lost, and the cytoplasm tended to spill out of the cell leading to cell death Fig. 5C. TEM micrographs of untreated MRSA M-4 were uniformly shaped with an undamaged structure of the inner membrane and an intact slightly waved outer surface. The periplasmic space was thin and had a uniform appearance with intact cell walls. The intracellular components displayed a homogeneous electron density Fig. 5D. After treatment with a sublethal dose of penicillin and ZA-S combination, some cells appeared as completely damaged and majority of the bacteria demonstrated strong evidence of membrane damage and distortion with greater roughness as compared to the control MRSA M-4. The walls of these cells were partially injured and the periplasmic space was thick and filled with electron-dense material from the cytosol and cells with apparently normal walls, but devoid of cytoplasmic contents Fig. 5E. Cells of MRSA M-4 that were subjected to a lethal dose of penicillin and ZA-S combination were commonly observed as lysed cells, but there were some unruptured cells which exhibited a great morphological change i.e., sleazy peripheral cell surface, hollow formation and cell disintegration which was also observed Fig. 5F.

### Cytotoxicity study

**Figure 6:**
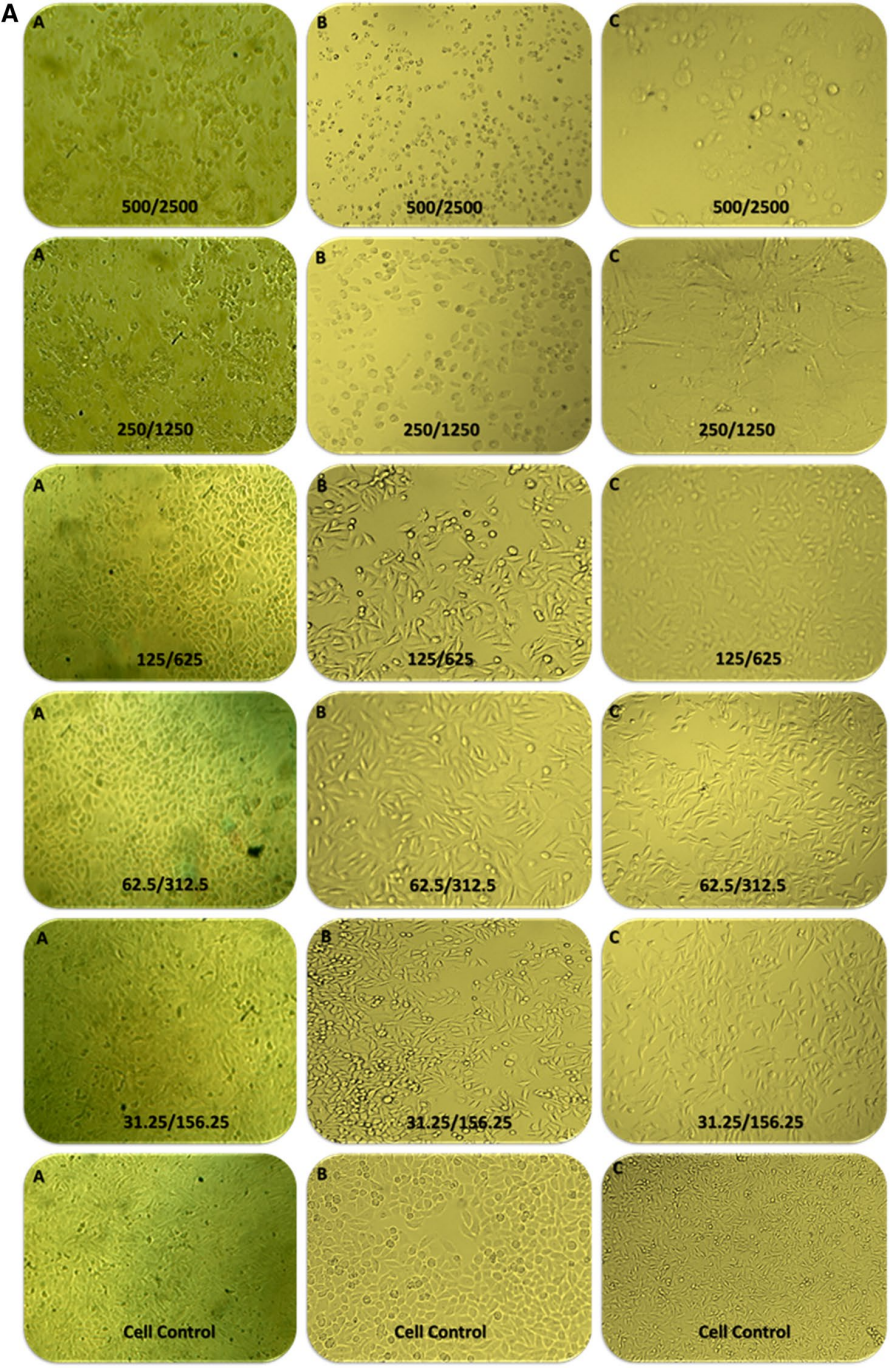

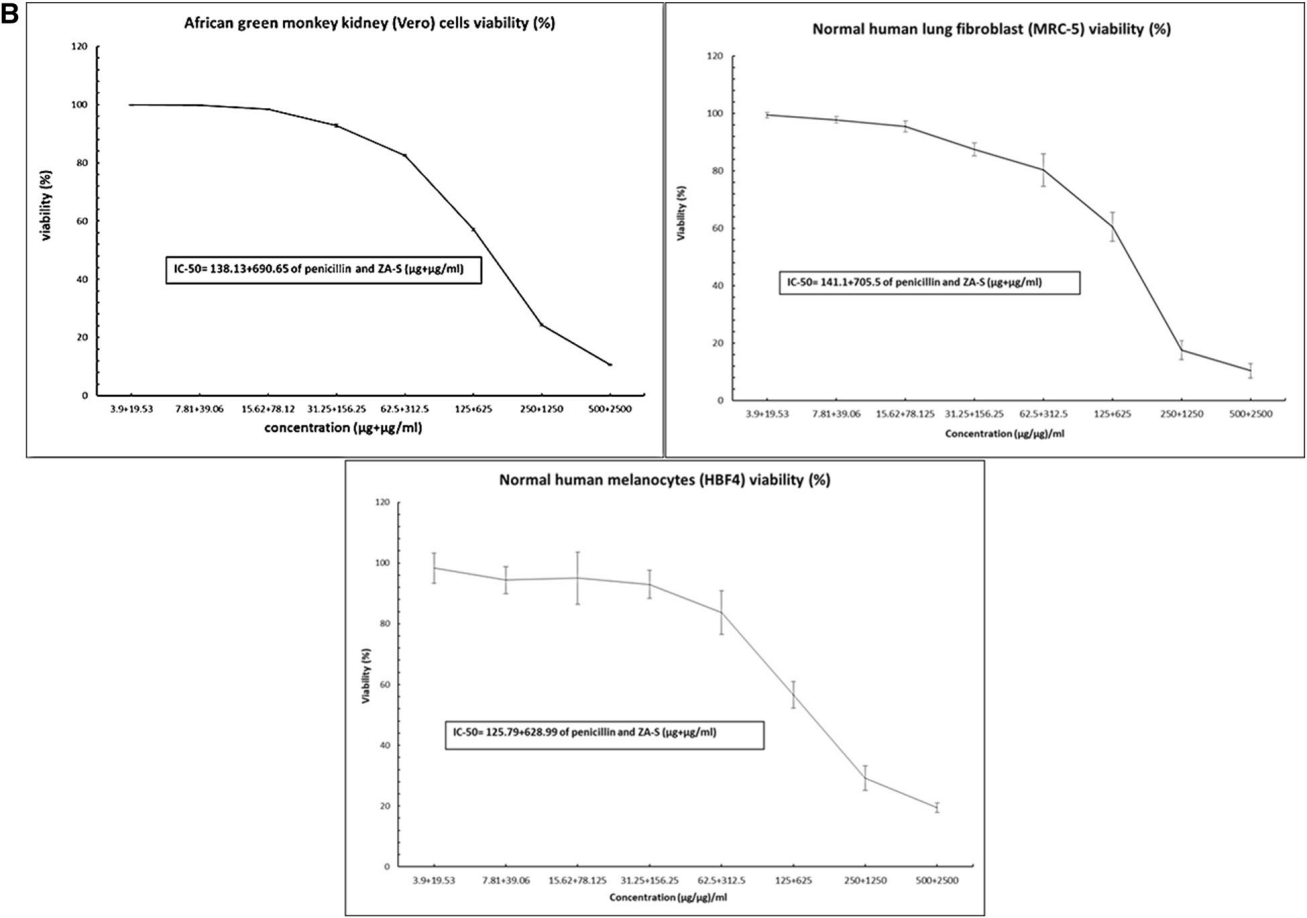
(**A**) Morphological observation of Vero cells (A), MRC-5 (B), and HBF4 (C) treated with different concentrations of penicillin and ZA-S combination under an inverted microscope. (**B**) Cytotoxicity results of gradient concentrations of penicillin and ZA-S combination on Vero, MRC-5, and HBF4 cells using MTT assay.

The morphological observation of Vero, MRC-5 and HBF4 normal cell lines treated with the combination of penicillin and ZA-S at different ratios indicated that there were slight changes in cells morphology including enlargement and minor granulation in the three types of cells, **e**specially at high concentration ratio (500 + 2500 µg + µg/ml of penicillin and ZA-S, respectively) compared to control Fig. 6A. The MTT assay results referred to the significant effect of the combinations on cell viability at a high ratio starting from 500 + 2500 µg + µg/ml of penicillin and ZA-S, respectively. Concentration of the combination required for 50% cell inhibition (IC-50 values) was 138.13 + 690.65, 141.1 + 705.5 and 125.79 + 628.99 µg + µg/ml of penicillin and ZA-S respectively in the case of Vero, MRC-5 and HBF4 cells, respectively Fig. 6B.

## Discussion

In our study, the obtained clinical isolates were identified as *Staphylococcus aureus*. The identified isolates were classified as methicillin resistance (oxacillin resistance) when it was resistant to cefoxitin antibiotic as a surrogate for oxacillin using the disk diffusion method^44^. Also, these isolates were classified as multidrug-resistant bacteria because they were resistant to at least one antibiotic in three or more antibiotic categories^44^. Based on the antibiotic profiles, *Staphylococcus aureus* ATCC-25923 was used as the standard strain that exhibited inhibition zone diameters inside the quality control ranges of sensitivity^45^.

MRSA is the most common multidrug-resistant gram-positive organism causing healthcare-associated infections (HAI). Therefore, MRSA remains an important goal for infection control and prevention measures^4^.

Saponin fraction of *Zygophyllum album* exhibited considerable antibacterial activity. This activity may attribute to their contents of active compounds which has a broad spectrum of biological and pharmacological compounds. Our results confirmed that *Zygophyllum album* separated saponins possess the strongest antibacterial activity. Antibacterial activity of saponins from some plant sources has been already reported^46^. The antibacterial activity of the crude extract against all tested bacteria was smaller than the saponin fraction. This may be due to the low concentration of saponin in the crude extract.

Identifying secondary metabolites in plants using mass spectrometric techniques has been progressively applied as an accurate tool used for medicinal plants’ analysis^47^. The ultra-high-performance liquid chromatography-quadrupole time-of-flight mass spectrometry (UHPLC/QTOF-MS) technique is a modern approach in the chromatography field. It has different advantages such as being a fast, sensitive and high-resolution separation technique^48^. UHPLC/QTOF-MS provides an accurate analysis of different kinds of secondary metabolites with different polarities compared to standard LC methods. It can be considered a faster and much more sensitive reliable tool to identify secondary metabolites as compared to other conventional chromatography separation techniques^49^.

The MIC values of penicillin and ZA-S against MRSA seemed as high value compared to MIC values obtained in the case of *S. aureus* ATCC 25923. This may be attributed to the resistance genes that more likely could be responsible for the emergence of some bacterial isolates with high MIC values^50^. Although some papers reported the antimicrobial activity of essential oil of leaves and extract of *Zygophyllum album* against *S. aureus, S. epidermidis, E. coli, B. subtilis* and *Serratia marcescens*^51^. However, it is difficult to compare the data with the literature because several variables influence the results, such as the different chemical composition due to the environmental factors (such as geography, temperature, day length, nutrients, etc.) of the plants.

Combination therapy is the most commonly recommended empirical treatment for bacterial infections in an intensive care units where the combined therapy has numerous benefits that include treatment of mixed infections, the infection caused by specific causative organism, to increase antimicrobial activity, prevent the need for long term antibiotic use and prevent the emergence of multidrug-resistant bacteria^31^.

According to the results obtained from the combinations of penicillin and ZA-S, it is clear to report that checkerboard assay determined the concentration of each agent in the combination at which the synergy was done when the FIC index is ≤ 0.5. These synergistic combinations of penicillin and ZA-S lower the amount of both agents in the dosage (at least reduced to one-fourth of the corresponding MIC) where the MIC values of penicillin and ZA-S before combination were 250 and 1250 µg/ml respectively and reduced to 62.5 + 312.5, 62.5 + 156.25 and 62.5 + 78.125 (µg/µg)/ml of penicillin and ZA-S respectively in a synergistic combination. This activity may result from the double force of both penicillin and ZA-S together on the cell where saponins have detergent-like properties and might increase the permeability of bacterial cell membranes; this action might facilitate antibiotic influx through the bacterial cell membrane. Therefore, it may cause the enhancement of antibiotic entry and increase its concentrations at the place of antibiotic–microbe contact, and thus speed up the binding between microbes and antibiotics^52, 53^.

Time kill study not only gives the information about the nature of the combinations whether it is bactericidal or bacteriostatic but also the capability for detecting antimicrobial agent activity over time and it is a suitable method for assessing changes in the antimicrobial agent activity^54^. In our study, although the combination at 62.5 + 156.25 (µg + µg)/ml of penicillin and ZA-S respectively suppressed the growth of MRSA M-4 during all time intervals of the experiment, the reduction of CFU count was not exceeded than − 1.8 in comparison with the CFU count of the initial inoculum. Moreover, CFU count in the case of the treated cells with the combination at 62.5 + 78.125 (µg + µg)/ml of penicillin and ZA-S respectively approximately was not affected during all time intervals. According to Lorian^34^ who suggests that whether an agent reduces the bacterial count of a pathogen by 3 log10 within 24 h of incubation in liquid media, the agent is classified as bactericidal for that particular pathogen. While bacteriostatic agent was defined as causing a decrease of < 3 log10 CFU/ml compared with the initial inoculum, the combination action in the previous cases is considered bacteriostatic. On the other side, the combination at 62.5 + 312.5 (µg + µg)/ml of penicillin and ZA-S displayed a marked increase in antibacterial activity and reduced the CFU count number by − 3.7. So, this combination is considered bactericidal. Based on the results of this experiment, it is possible to conclude that the penicillin has been strengthened by ZA-S to kill or inhibit the cells of MRSA in concentration-dependent behavior.

Scanning and transmission electron microscopy studies were performed to evaluate the effects and changes that can occur to cells after their treatment with the combinations of penicillin and ZA-S at lethal and sublethal doses. The results obtained from micrographs of SEM and TEM were compatible, whereas in all cases, there is an important observation that the affected cells usually large in their size and are injured in walls. This may be due to the blocking of cell wall formation through the cell duplication stage by the effect of penicillin coupled with ZA-S. Elliott et al.^55^ reported that it is probable that the cells are markedly affected when exposed to penicillin in the phase of growth.

A cytotoxicity study using MTT assay and morphological changes observation indicated that there was no cytotoxic effect that occurred by the synergistic combination at the concentration having bactericidal activity. This may be owing to the concentration of penicillin in our combination lies very close to the standard concentration in cell culture media approved by American Type Culture Collection (ATCC) (50 to 100 I.U./ml that is equivalent 29.95 to 59.9 µg/ml)^56, 57^ On the other hand, the concentration of ZA-S in our combination was 78.12 µg/ml and this concentration is considered far away from the IC_50_ of *Zygophyllum album* against normal cell human skin fibroblast (WS1) (≥ 200 µg/ml)^58^.

## Conclusion

Different metabolites were isolated from the *Zygophyllum album* such as saponin, phenolic and steroidal fractions. According to the results, we conclude that Ursane-type saponins of *Zygophyllum album* has activity against different clinical isolates of MRSA. The results described herein provide significant enhancement of penicillin activity against MRSA if it is combined with saponin fraction of *Zygophyllum album* under the conditions implemented in the current study. Besides, synergistic combinations tested in this work exhibit antibacterial effects at non-toxic concentrations for different normal cells. Despite our findings in this research, further studies are required on an animal model to confirm the anti-MRSA activity observed in vitro.

## Supplementary Information

Supplementary Information.

## References

[1] Ventola CL (2015). The antibiotic resistance crisis: part 1: causes and threats. Pharm. Therap..

[2] Tyers M, Wright GD (2019). Drug combinations: a strategy to extend the life of antibiotics in the 21st century. Nat. Rev. Microbiol..

[3] Bueno J (2016). Antimicrobial adjuvants drug discovery, the challenge of avoid the resistance and recover the susceptibility of multidrug-resistant strains. J. Microb. Biochem. Technol..

[4] Kramer T, Schröder C, Behnke M (2019). Decrease of methicillin resistance in *Staphylococcus aureus* in nosocomial infections in Germany—a prospective analysis over 10 years. J. Infect..

[5] Abiramasundari P, Priya V, Jeyanthi G, Gayathri D (2011). Evaluation of the antibacterial activity of cocculus hirsutus. J. Drugs Med..

[6] Bhardwaj M, Singh B, Sinha D, Kumar V, Prasanna VO (2016). Potential of herbal drug and antibiotic combination therapy: a new approach to treat multidrug-resistant bacteria. Pharm. Anal. Acta.

[7] Worthington RJ, Melander C (2013). Combination approaches to combat multidrug-resistant bacteria. Trends Biotechnol..

[8] Olajuyigbe OO, Afolayan AJ (2015). In vitro synergy and time-kill assessment of interaction between kanamycin and metronidazole against resistant bacteria. Trop. J. Pharm. Res..

[9] Saleh NA, El-Hadidi MN (1977). An approach to the chemosystematics of the *Zygophyllaceae*. Biochem. Syst. Ecol..

[10] Täckholm V, Drar M (1954). Flora of Egypt. Bull. Fac. Sci. Egypt. Univ..

[11] El-Wahab RHA, Zaghloul MS, Kamel WM, Moustafa A (2008). Diversity and distribution of medicinal plants in North Sinai, Egypt. Afr. J. Environ. Sci. Technol..

[12] Hassanean H, Desoky E, El-Hamouly M (1993). Quinovic acid glycosides from *Zygophyllum album*. Phytochemistry.

[13] Hassanean H, El-Hamouly M, El-Moghazy S, Bishay D (1993). 14-Decarboxyquinovic and quinovic acid glycosides from *Zygophyllum album*. Phytochemistry.

[14] Belguidoum M, Dendougui H, Kendour Z (2015). In vitro antioxidant properties and phenolic contents of *Zygophyllum album* L. from Algeria. J. Chem. Pharm. Res..

[15] Moghannem SA, El-Sherbiny GM, Kalaba MH (2017). Isolation and identification of *Streptomyces baarnensis* MH-133 produce bioactive metabolite inhibiting multidrug-resistant bacteria (MDRB). World J. Pharm. Med. Res..

[16] Patel J, Cockerill F, Eliopoulos G (2017). M100 Performance Standards for Antimicrobial Susceptibility Testing.

[17] Parvez MM, Rahman MA, Molla MK, Akter A (2012). Compound isolation and purification by chromatographic method of stem bark of *Anisoptera scaphula* (Roxb.). Int. J. Pharma Res. Rev..

[18] Sarker SD, Nahar L (2012). An Introduction to Natural Products Isolation. Natural Products Isolation.

[19] Hostettmann K, Marston A (1995). Saponins.

[20] Woof J, Pierce J (1967). Separation of complex mixtures of polyhydroxy phenols on columns of Sephadex. J. Chromatogr. A.

[21] Kantz K, Singleton V (1990). Isolation and determination of polymeric polyphenols using Sephadex LH-20 and analysis of grape tissue extracts. Am. J. Enol. Vitic..

[22] Ichihara K, Fukubayashi Y (2010). Preparation of fatty acid methyl esters for gas-liquid chromatography. J. Lipid Res..

[23] Kamal R, Yadav R, Sharma J (1993). Efficacy of the steroidal fraction of fenugreek seed extract on fertility of male albino rats. Phytother. Res..

[24] Dinan L, Harmatha J, Lafont R (2001). Chromatographic procedures for the isolation of plant steroids. J. Chromatogr. A.

[25] Parekh J, Chanda S (2007). Antibacterial and phytochemical studies on twelve species of Indian medicinal plants. Afr. J. Biomed. Res..

[26] Fayek NM, Farag MA, Abdel Monem AR, Moussa MY, Abd-Elwahab SM, El-Tanbouly ND (2019). Comparative metabolite profiling of four citrus peel cultivars via ultra-performance liquid chromatography coupled with quadrupole-time-of-flight-mass spectrometry and multivariate data analyses. J. Chromatogr. Sci..

[27] Tsugawa H, Cajka T, Kind T (2015). MS-DIAL: data-independent MS/MS deconvolution for comprehensive metabolome analysis. Nat. Methods.

[28] Clinical, Institute LS (2009). Performance Standards for Antimicrobial Susceptibility Testing of Anaerobic Bacteria: Informational Supplement.

[29] Sopirala MM, Mangino JE, Gebreyes WA (2010). Synergy testing by Etest, microdilution checkerboard, and time-kill methods for pan-drug-resistant *Acinetobacter baumannii*. Antimicrob. Agents Chemother..

[30] Isaei E, Mansouri S, Mohammadi F, Taheritarigh S, Mohammadi Z (2016). Novel combinations of synthesized ZnO NPs and ceftazidime: evaluation of their activity against standards and new clinically isolated *Pseudomonas aeruginosa*. Avicenna J. Med. Biotechnol..

[31] Joung DK, Choi SH, Kang OH (2015). Synergistic effects of oxyresveratrol in conjunction with antibiotics against methicillin-resistant *Staphylococcus aureus*. Mol. Med. Rep..

[32] Barry AL, Standards NCCLS (1999). Methods for Determining Bactericidal Activity of Antimicrobial Agents: Approved Guideline.

[33] Konaté K, Hilou A, Mavoungou JF (2012). Antimicrobial activity of polyphenol-rich fractions from *Sida alba* L. (Malvaceae) against cotrimoxazole-resistant bacteria strains. Ann. Clin. Microbiol. Antimicrob..

[34] Lorian V (2005). Antibiotics in Laboratory Medicine.

[35] Abd-Elnaby H, Abo-Elala G, Abdel-Raouf U, Abd-elwahab A, Hamed M (2016). Antibacterial and anticancer activity of marine *Streptomyces parvus*: optimization and application. Biotechnol. Biotechnol. Equip..

[36] Payne JN, Waghwani HK, Connor MG (2016). Novel synthesis of kanamycin conjugated gold nanoparticles with potent antibacterial activity. Front. Microbiol..

[37] Helmy EA, Mekawey AA (2014). Envision of the microbial contact with mycosynthesized silver nanoparticles. Res. J. Pharm. Biol. Chem. Sci..

[38] Chen Y-T, Yuan Q, Shan L-T, Lin M-A, Cheng D-Q, Li C-Y (2013). Antitumor activity of bacterial exopolysaccharides from the endophyte *Bacillus amyloliquefaciens* sp. isolated from Ophiopogon japonicus. Oncol. Lett..

[39] Abdelnasser SM, Yahya SM, Mohamed WF (2017). Antitumor exopolysaccharides derived from novel marine *Bacillus*: isolation, characterization aspect, and biological activity. Asian Pac. J. Cancer Prevent. APJCP.

[40] AAT Bioquest, Inc. (2020, December 14). Quest Graph™ IC50 Calculator (v.1).. Retrieved from https://www.aatbio.com/tools/ic50-calculator-v1

[41] Pöllmann K, Gagel S, Elgamal MHA, Shaker KH, Seifert K (1997). Triterpenoid saponins from the roots of *Zygophyllum* species. Phytochemistry.

[42] Elgamal MHA, Shaker KH, Pöllmann K, Seifert K (1995). Triterpenoid saponins from *Zygophyllum* species. Phytochemistry.

[43] Ibrahim N (1997). Saponinfrom *Zygophyllum album* and biological investigation. Egypt. J. Pharm. Sci..

[44] CLSI (2018). Performance Standards for Antimicrobial Susceptibility Testing. 28h ed. CLSI Supplement M100.

[45] Merli M, Lucidi C, Di Gregorio V, Falcone M, Giannelli V, Lattanzi B (2015). The spread of multi-drug resistant infections is leading to an increase in the empirical antibiotic treatment failure in cirrhosis: a prospective survey. PLoS ONE.

[46] Avato P, Bucci R, Tava A (2006). Antimicrobial activity of saponins from *Medicago* sp.: structure-activity relationship. Phytother. Res. Int. J. Devoted Pharmacol. Toxicol. Eval. Nat. Prod. Deriv..

[47] Wolfender J-L, Rudaz S, Hae Choi Y, Kyong KH (2013). Plant metabolomics: from holistic data to relevant biomarkers. Curr. Med. Chem..

[48] Khan H, Ali J (2015). UHPLC/Q-ToF-MS technique: introduction and applications. Lett. Org. Chem..

[49] Hanhineva K, Soininen P, Anttonen MJ (2009). NMR and UPLC-qTOF-MS/MS characterization of novel phenylethanol derivatives of phenylpropanoid glucosides from the leaves of strawberry (Fragaria × ananassa cv. Jonsok). Phytochem. Anal..

[50] Japoni A, Ziyaeyan M, Jmalidoust M (2010). Antibacterial susceptibility patterns and cross-resistance of methicillin-resistant and sensitive *Staphylococcus aureus* isolated from the hospitalized patients in Shiraz Iran. Braz. J. Microbiol..

[51] Belmimoun A, Meddah B, Meddah A, Sonnet P (2016). Antibacterial and antioxidant activities of the essential oils and phenolic extracts of *Myrtus communis* and *Zygophyllum album* from Algeria. J. Fundam. Appl. Sci..

[52] Allahverdiyev AM, Kon KV, Abamor ES, Bagirova M, Rafailovich M (2011). Coping with antibiotic resistance: combining nanoparticles with antibiotics and other antimicrobial agents. Exp. Rev. Anti-infect. Therapy.

[53] Khan, M. I., Ahmed, A., Shin, J. H., Baek, J. S., Kim, M. Y. & Kim, J. D. Green tea seed isolated saponins exert antibacterial effects against various strains of Gram-positive and Gram-negative bacteria, a comprehensive study in vitro and in vivo. *Evid. Based Complement. Altern. Med.* **2018**, 3486106 (2018). 10.1155/2018/3486106 PMC6287149 30598684

[54] Appiah, T., Boakye, Y. D. & Agyare, C. Antimicrobial activities and time-kill kinetics of extracts of selected ghanaian mushrooms. *Evid. Based Complement. Altern. Med.* **2017**, 4534350 (2017). 10.1155/2017/4534350 PMC5682094 29234399

[55] Elliott T, Greenwood D, Rodgers F, O'Grady F (1979). The response of *Staphylococcus aureus* to benzylpenicillin. Br. J. Exp. Pathol..

[56] Humphrey J, Mussett M, Perry W (1953). The second international standard for penicillin. Bull. World Health Organ..

[57] Yazici H, Alpaslan E, Webster TJ (2015). The role of dextran coatings on the cytotoxicity properties of ceria nanoparticles toward bone cancer cells. JOM.

[58] Ksouri WM, Medini F, Mkadmini K (2013). LC–ESI-TOF–MS identification of bioactive secondary metabolites involved in the antioxidant, anti-inflammatory and anticancer activities of the edible halophyte *Zygophyllum album* Desf. Food Chem..

