# Supplementary material for: New combination approaches to combat methicillin-resistant Staphylococcus aureus (MRSA)
Source: Sci Rep. 2021 Feb 19;11:4240. doi: 10.1038/s41598-021-82550-4 (PMC7896049; doi:10.1038/s41598-021-82550-4)
Supplement: Supplementary file 1 — Supplementary Information. [file 41598_2021_82550_MOESM1_ESM.docx]

**New Combination Approaches to Combat Methicillin-Resistant *Staphylococcus aureus* (MRSA)**

**Mohamed H. Sharaf^1^, Gamal M. El-Sherbiny^1^, Saad A. Moghannem^1,^ Mohamed Abdelmonem^2^, Islam A. Elsehemy^3^, Ahmed M. Metwaly^4^, Mohamed H. kalaba^1^,**

**^1^** Botany and Microbiology Department, Faculty Science, Al-Azhar University, Cairo, Egypt. **^2^**Clinical Laboratory, Stanford Healthcare, USA.

**^3^**Chemistry of natural and microbial products, National Research Centre, Giza, Egypt.

**^4^** Pharmacognosy department, faculty of Pharmacy, Al-Azhar University, Cairo, Egypt.

**Corresponding author: Gamal M. El-Sherbiny**

Email: **gamalelsherbiny1970@yahoo.com** **ORCID ID 0000-0003-3968-0536**

Tel.: +201064665699

**Address:**

Botany and Microbiology Department, Faculty Science, Al-Azhar University, Cairo, Egypt

1. **Identification of clinical isolates using vitek2 system**

| **Well** | **Test** | **Code** | **Results** | | | |
| --- | --- | --- | --- | --- | --- | --- |
|  |  |  | **M-1** | **M-2** | **M-3** | **M-4** |
| **2** | **D-Amygdalin** | **AMY** | - | - | - | - |
| **4** | **Phosphatidylinositol Phospholipase** | **PIPLC** | - | + | - | - |
| **5** | **D-Xylose** | **Dxyl** | - | - | - | - |
| **8** | **Argnine Dihydrolase** | **ADH1** | + | + | + | + |
| **9** | **Beta-galactosidase** | **BGAL** | - | - | - | - |
| **11** | **Alpha-glucosidase** | **AGLU** | + | + | + | - |
| **13** | **Ala-phe-pro- Arylamidase** | **APPA** | - | - | - | - |
| **14** | **Cyclodextrin** | **CDEX** | - | - | - | - |
| **15** | **L- Aspartate Arylamidase** | **AspA** | - | - | - | + |
| **16** | **Beta galactopyranosidase** | **BGAR** | - | - | - | + |
| **17** | **Alpha-Mannosidase** | **AMAN** | - | - | - | + |
| **19** | **Phosphatase** | **PHOS** | + | + | + | - |
| **20** | **Leucine Arylamidase** | **LeuA** | - | - | - | - |
| **23** | **L-Prolin Arylamidase** | **ProA** | - | - | - | - |
| **24** | **(Beta- Glucuronidase)** | **BGURr** | - | - | - | - |
| **25** | **Alpha- galactocidase** | **AGAL** | - | - | - | - |
| **26** | **L-Pyrrolidonyl-Arylamidase** | **PyrA** | + | + | + | + |
| **27** | **Beta Glucuronidase** | **BGUR** | - | - | - | + |
| **28** | **Alanine Arlamidase** | **ALaA** | - | - | - | + |
| **29** | **Tyrosine Arylamidase** | **TyrA** | - | - | - | + |
| **30** | **D-Sorbitol** | **dSOR** | - | - | - | - |
| **31** | **Urease** | **URE** | **-** | **-** | **-** | + |
| **32** | **Polymixin B resistance** | **POLYB** | - | + | + | + |
| **37** | **D-Galactose** | **dGAL** | - | + | - | + |
| **38** | **D-Ribose** | **dRIB** | + | + | + | + |
| **39** | **L-Lactate Alkalinization** | **iLATK** | + | + | + | - |
| **42** | **Lactose** | **LAC** | - | - | - | - |
| **44** | **N- Acetyle-D- Glucosamine** | **NAG** | + | + | + | + |
| **45** | **D-maltose** | **dMAL** | + | + | + | + |
| **46** | **Bacitracine Resistance** | **BACi** | - | + | + | + |
| **47** | **Novobiocin Resistance** | **NOVO** | + | - | + | + |
| **50** | **Growth in 6.5% NaCl** | **NC6.5** | + | + | + | - |
| **52** | **D-mannitol** | **dMAN** | - | - | - | - |
| **53** | **D-Mannose** | **dMNE** | - | - | - | - |
| **54** | **Methyl-B-D-Glucopyranoside** | **MBdG** | - | - | - | + |
| **56** | **Pullulan** | **PUL** | - | - | - | + |
| **57** | **D- Raffinose** | **dRAF** | - | - | - | + |
| **58** | **O/129 resistance** | **O129R** | + | + | + | - |
| **59** | **Salicin** | **SAL** | - | - | - | + |
| **60** | **Sucrose** | **SAC** | + | + | + | + |
| **62** | **D-Trehalose** | **dTRE** | + | + | + | - |
| **63** | **Arginine Dihydrolase2** | **ADH2S** | - | - | - | + |
| **64** | **Optochin resistance** | **OPTO** | + | + | + | - |

**1-Antibiotic profile of clinical isolates**
